# Effect of Process Parameters on Organic Micro Patterns Fabricated on a Flexible Substrate Using the Near-Field Electrohydrodynamic Direct-Writing Method

**DOI:** 10.3390/mi10050287

**Published:** 2019-04-27

**Authors:** Jianzhou Chen, Ting Wu, Libing Zhang, Peng Li, Xiaowei Feng, Dazhen Li

**Affiliations:** College of Mechanical and Electrical Engineering, Jiaxing University, Jiaxing 314001, China; jzchen1018@gmail.com (J.C.); lipeng10.james@gmail.com (P.L.); fengxiaowei1014@gmail.com (X.F.); lidz0617@gmail.com (D.L.)

**Keywords:** electrohydrodynamics, near-field direct-writing method, process parameter, organic micro pattern, organic conductive polymer, PEDOT:PSS

## Abstract

A micro pattern is a key component of various functional devices. In the present study, using the poly(3,4-ethylenedioxythiophene):poly(styrenesulfonate) (PEDOT:PSS) mixed material as the direct-writing solution and photographic paper as the flexible insulating substrate, the organic micro patterns of various shapes, such as the curve of the second-order self-similar structure, the helical curve, and the wave curve, were fabricated on the flexible insulating substrate by using the near-field electrohydrodynamic direct-writing method. The effects of process parameters, such as the applied voltage, direct-writing height, flow rate of the injection system, and moving velocity of the substrate, on the width and the conductivity of the organic micro patterns were studied in the near-field electrohydrodynamic direct-writing process. The results show that the width of an organic micro pattern increases with the increase of the applied voltage of the high-voltage power supplier and the flow rate of the injection system under the condition where the three other process parameters remained constant, respectively, while the width of an organic micro pattern decreases with the increase of the direct-writing height and the moving velocity of the flexible substrate, respectively. The fabricated organic microcircuit patterns of the natural drying in air at room temperature were tested by a thin film thermoelectric tester at a detection temperature. The results show that the conductivity of a fabricated organic micro pattern decreases with the increase of the electric field intensity, while the effect of moving velocity and the flow rate on the conductivity is small under the condition where the three other process parameters remained constant.

## 1. Introduction

A micro pattern is a key component of various functional devices, such as a solar cell [1], biochip [2], wearable device [3], flexible electronics [4], organic light-emitting diode [5], thin film transistor [6], electronic skin [7], electronic newspaper [8], flexible electronic display [9], etc. Recently, these functional devices have attracted much attention from researchers. Lithography is the main technology used to fabricate micro patterns. Gather et al. [10] fabricated the first full-color polymer organic light-emitting diode (OLED) display by direct photolithography. The technology requires the use of extreme ultraviolet lithography to achieve the limit of physical resolution, which incurs high manufacturing costs. Han et al. [11] used nanoimprint technology to fabricate an anti-reflective layer to enhance the performance of solar cells. Guo [12] reported that the imprint lithography technique has been used to fabricate functional devices of polymer materials. However, the imprint lithography technique still has some drawbacks in that the adhesion between the template and the polymer often leads to defects in fabricating micro patterns.

In recent years, as a new micro-structure manufacturing technology, electrohydrodynamic (EHD) inkjet printing technology has received increasing attention. Khan et al. [13] used the multi-nozzle EHD inkjet printing method to fabricate copper conductive micro-tracks. The method overcomes the limitations of low production efficiency of EHD inkjet printing technology. An et al. [14] used EHD inkjet printing technology to print three-dimensional structures. This technology can realize multi-function inkjet printing. Kim et al. [15] fabricated a stretchable metal oxide semiconductor transistor by using the EHD inkjet printing method. The fabricated transistor has high resolution. Zhang et al. [16] used EHD inkjet printing technology to manufacture sub-micrometer and three-dimensional structures. Their proposed method utilizes a simple process and is low cost. Jinke et al. [17] fabricated the microstructure of conductive polymer material by using the EHD printing method. The method is conducive to the application of organic materials in flexible and wearable devices. Kim et al. [18] fabricated high-resolution organic small molecular light-emitting diodes using the EHD inkjet printing method. Xu et al. [19] used EHD inkjet printing technique to fabricate a rechargeable lithium ion battery. However, the charged jet from the top of the Taylor cone generates whipping under the action of charge repulsion force and inhomogeneous electric field force in the process of the EHD inkjet printing, which affects the positioning accuracy of the micro pattern. As noted above, a micro pattern is the key part of functional devices, and a positioning error in a micro pattern has an important effect on the performance of functional devices.

In order to reduce the effect of whipping on the deposited pattern in the EHD inkjet printing process, the jet height between the nozzle and the flexible substrate is shortened and the deposited micro pattern is completed before the instability and whipping of the charged jet occurs, which results in near-field EHD direct-writing (NFEDW) technology. As a novel noncontact printing technology, Zheng et al. [20] used NFEDW technology to construct a stretchable micro pattern. This technology can effectively improve the positioning accuracy of the deposited micro pattern. Duan et al. [21] fabricated the flexible piezoelectric device on polydimethylsiloxane (PDMS) substrate by using the NFEDW method. This method can be used to fabricate flexible sensors. Ali et al. [22] used NFEDW technology to fabricate a stretchable photo sensor. The fabricated sensor can be used in wearable electronic devices. Fuh et al. [23] presented near-field electrospinning technology to fabricate a self-powered pressure sensor. This technology can be used to construct a three-dimensional structure, which has broad application prospects in biomedicine and wearable electronics. Lei et al. [24] verified that NFEDW technology has excellent ability to fabricate all kinds of micro patterns, such as a linear segment, a cross grid, an arc line, a curve, a bead-on-string structure, etc. Thus, the technology can help to accurately develop a micro pattern. Zhang et al. [25] reported that the technology played an important role in the fabrication of a flexible electronic micro pattern. The main micro-patterning techniques are compared and shown in Table 1. However, compared with traditional EHD inkjet printing technology, NFEDW technology is still limited by technical difficulties in many applications, such as with respect to the effect of process parameters on the organic micro patterns of poly(3,4-ethylenedioxythiophene):poly(styrenesulfonate) (PEDOT:PSS) material fabricated on a flexible substrate.

PEDOT:PSS is an organic, conductive polymer material, which is widely used in the field of flexible electronics and wearable devices. In this paper, the effects of process parameters, such as the applied voltage of the high-voltage power supplier, direct-writing height between the nozzle and the flexible substrate, moving velocity of the flexible substrate, and flow rate of the injection system, on the width and the conductivity of the fabricated micro pattern were studied by using the self-developed NFEDW experimental setup with PEDOT:PSS mixed material as the direct-writing solution and flexible insulating photographic paper as the flexible substrate.

## 2. Materials and Methods

### 2.1. Materials

Poly(3,4-ethylenedioxythiophene):poly(styrenesulfonate) (Heraeus PH1000, Heraeus, Hanau, Germany) was used to prepare the organic conductive polymer mixed solution for the experiment. DMSO (Chinasun Specialty Products Co., Ltd., Changshu, China) was a supporting solvent. The PEDOT:PSS solution was mixed with DMSO solution at a volume ratio of 1:0.2. Triton X-100 (Sinopharm Chemical Reagent Co., Ltd., Shanghai, China) was added as a surfactant to the formulated solution at a mass ratio of 0.1% to reduce the surface tension of the solution, which helped to form the direct-writing jet. The three solutions were mixed and stirred in a magnetic blender (Asone RS-IDN, Osaka, Japan) at 600 rpm for 10 h to complete the solution preparation, and then, the mixed solution was used for the NFEDW experiment. Photographic paper (Epson A4) was used as a flexible insulating substrate, which could produce bending deformation.

### 2.2. The Near-Field Electrohydrodynamic Direct-Writing (NFEDW) Method

**Figure 1:**
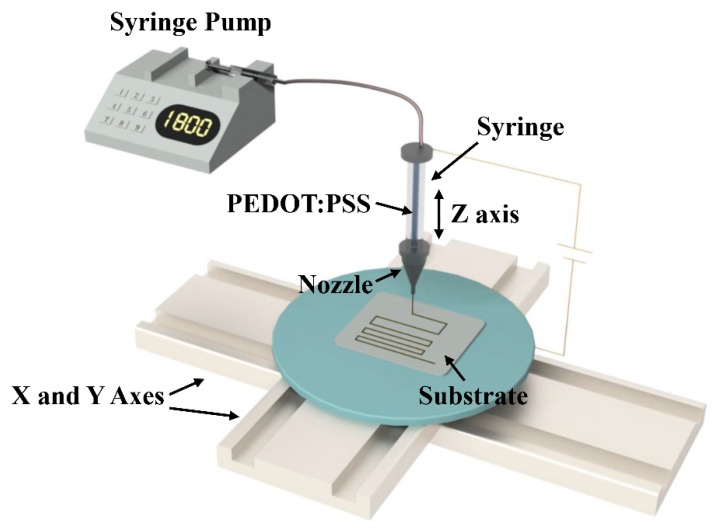
The schematic diagram of the near-field electrohydrodynamic direct-writing (NFEDW) method. PEDOT:PSS—poly(3,4-ethylenedioxythiophene):poly(styrenesulfonate).

The schematic diagram of the NFEDW method, which consists of the injection system, the high-voltage power supplier, and the motion platform, is illustrated in Figure 1. The injection system consists of a syringe pump, a syringe, and a nozzle. The motion platform consists of the X–Y motion platform and Z-axis. The high-voltage power supplier is connected between the nozzle and the substrate, which forms the electrostatic field. Under the action of the electric field, moving charges accumulate on the surface of the liquid, and Coulomb force generates shear stress on the surface of the liquid, which causes the liquid to form a Taylor cone at the top of the nozzle. With the increase of the electric field intensity between the nozzle and the substrate, the electric field force overcomes the surface tension of the liquid and generates the direct-writing jet at the top of the Taylor cone. At the same time, the X–Y motion platform carries out the movement, and the direct-writing pattern can be fabricated. The direct-writing height is adjusted by the z-axis motion, which is usually less than 5 mm. This method can realize the high-precision positioning of the direct-writing micro pattern.

The NFEDW method involves the coupling of multiple physical fields, such as the flow field, the electric field, and so on. The flow state of the direct-writing solution is affected by the electric field, and the electric field is also affected by the flow state of the charged solution. The control equations of the mathematical model for the coupling between the electric field and the flow field include the electrodynamics control equation and the fluid dynamics control equation of the direct-writing solution. For an incompressible and viscous solution, the electrodynamics equation can be expressed as follows

(1) $$\nabla E=\frac{Q}{\varepsilon }$$

(2) E=−∇ϕ

(3) $$\frac{\partial Q}{\partial t}+\nabla \cdot j=0$$

where *E* is the electric field intensity, *Q* is the charge density, *ε* is the permittivity, *ϕ* is the electric potential, *j* is the current density, and *t* is time.

**Figure 2:**
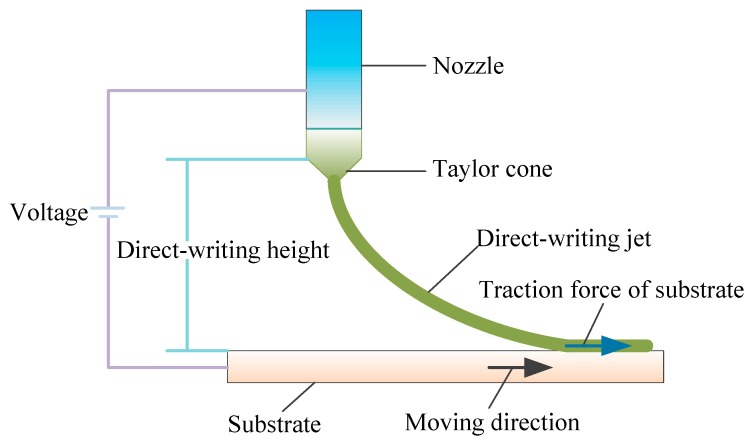
The direct-writing jet model of the NFEDW method.

The NFEDW method is used to write the viscous polymer solution directly, and the direct-writing jet model, which is subjected to the pressure, the electrostatic force, the traction force of the substrate, the surface tension, the gravity, etc., is shown in Figure 2. According to the Navier–Stokes equation, the fluid motion equation can be expressed as follows

(4) ∇⋅U=0

(5) $$\rho \frac{dU}{dt}=-\nabla P+\mu \nabla^{2}U+F+F_{s}+F_{e}+P_{f}$$

where U is the flow rate of the direct-writing solution, *ρ* is the density of the direct-writing solution, P is the pressure of the direct-writing solution, *μ* is the viscosity of the direct-writing solution, F denotes the gravity of the direct-writing solution, $F_{s}$ denotes the surface tension of the direct-writing solution, $F_{e}$ denotes the electric field force, and $P_{f}$ denotes the traction force of the substrate.

According to the Laudau theory, the electric field force of the direct-writing solution can be expressed as follows

(6) $$F_{e}=QE-\frac{1}{2}E^{2}\nabla \varepsilon +\frac{1}{2}\nabla \left[\rho \left(\frac{d\varepsilon }{d\rho }\right)E^{2}\right]$$

where $F_{e}$ denotes the electric field force, E denotes the electric field intensity, QE denotes Coulomb force, $\frac{1}{2}E^{2}\nabla \varepsilon$ is the bound charge force, and $\frac{1}{2}\nabla \left[\rho \left(\frac{d\varepsilon }{d\rho }\right)E^{2}\right]$ is the force caused by the density change of the direct-writing solution.

Therefore, in the NFEDW process, the direct-writing jet of the same solution is affected by some process parameters, such as the applied voltage, direct-writing height, flow rate of the injection system, and moving velocity of the substrate, which affects the morphology and characteristics of the micro pattern. The effect of process parameters on the direct-writing micro pattern was studied using an experimental method, and control of the direct-writing micro pattern in a viscous polymer solution was realized.

### 2.3. Experimental Setup

**Figure 3:**
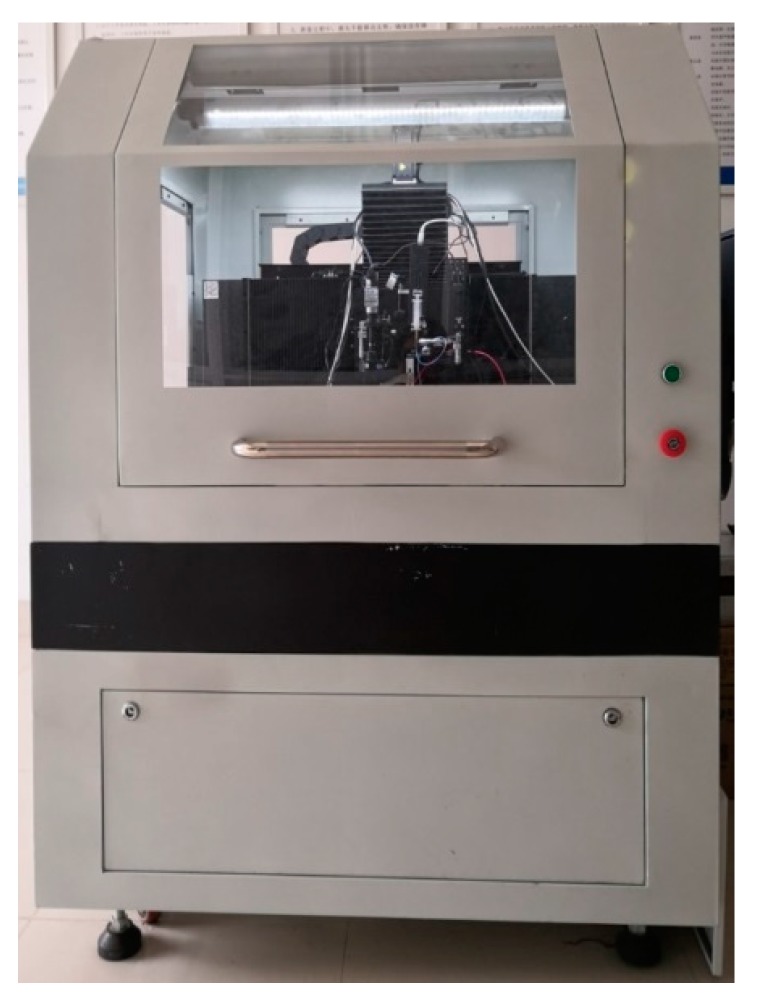
The self-developed experimental setup of the NFEDW method.

The experimental setup consisted of a motion platform, an injection system, and a high-voltage power supplier (DW-P303-2ACF1, Dongwen Inc., Tianjin, China). The motion platform consisted of the X-axis, Y-axis, and Z-axis. The X-axis and Y-axis were composed of the AC servo linear motor (HIWIN, Taichung, Taiwan), linear guide rail (HIWIN), and linear grating ruler (Renishaw, London, UK), respectively, and the Z-axis was composed of the AC servo motor (Panasonic, Tokyo, Japan), ball screw (HIWIN), linear guide rail (HIWIN), and linear grating ruler (Renishaw). The injection system consisted of a syringe pump (TJ-1A- Micro Flow Rate, Longer Precision Pump Co., Ltd., Baoding, China), syringe, and nozzle. The nozzle was a stainless-steel nozzle, which was used to generate the direct-writing jet, with an inner diameter of 200 µm and an outer diameter of 410 µm. The self-developed experimental setup of the NFEDW method is shown in Figure 3.

## 3. Results and Discussions

### 3.1. Fabricating Organic Micro Patterns on the Flexible Substrate

**Figure 4:**
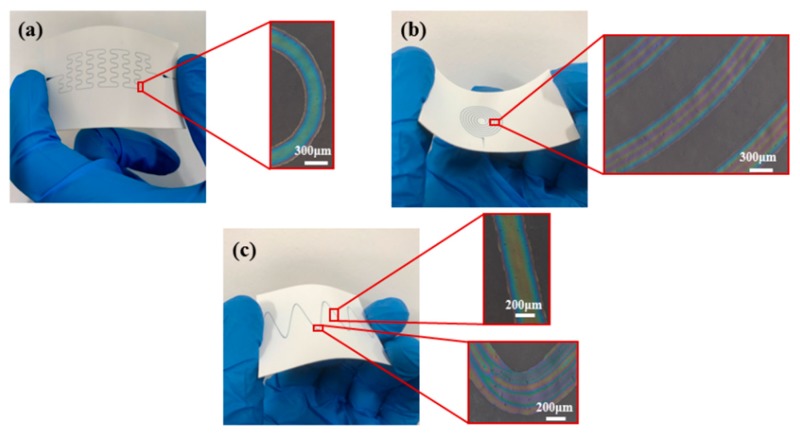
Fabricating organic micro patterns of various shapes on the flexible insulating substrate. (**a**) The organic micro pattern of the second-order self-similar structure curve. (**b**) The organic micro pattern of the helical curve. (**c**) The organic micro pattern of the wave curve.

The photographic paper was fixed on the motion platform and placed underneath the direct-writing nozzle. The direct-wring height between the nozzle and the flexible substrate was adjustable by the Z-axis of the motion platform. Though the movement of the motion platform, the PEDOT:PSS mixed solution was deposited on the photographic paper according to the preset micro pattern in the control system of the NFEDW setup under the electric field force applied by the high-voltage power supplier. Micro patterns of various shapes, which are shown in Figure 4, could be fabricated with the PEDOT:PSS mixed solution as the direct-writing solution by using the self-developed experimental setup of the NFEDW method. The curve of the second-order self-similar structure was fabricated under the conditions of the applied voltage of 2 kV, direct-writing height of 0.15 mm, moving velocity of 8 mm/s, and flow rate of 125 nL/min. The average width of the organic micro pattern was about 325 µm, which is shown in Figure 4a. The helical curve was fabricated under the conditions of the applied voltage of 2 kV, direct-writing height of 0.15 mm, moving velocity of 5.5 mm/s, and flow rate of 125 nL/min. The average width of the organic micro pattern was about 400 µm, which is shown in Figure 4b. The wave curve was fabricated under the conditions of the applied voltage of 2 kV, direct-writing height of 0.2 mm, moving velocity of 10 mm/s, and flow rate of 125 nL/min. The average width at corner of the wave curve was about 448 µm, while the average width of the other parts was about 350 µm, which is shown in Figure 4c. The movement distance at the corner decreased, so the width of the micro pattern increased. To analyze the uniformity and continuity of the organic micro patterns fabricated on the flexible insulating substrate, the deposited organic micro patterns were observed on an inverted metallographic microscope (DMI3000M, Leica, Wetzlar, Germany). The partially magnified curve of the second-order self-similar structure is shown in Figure 4a. The partially magnified helical curve is shown in Figure 4b. The partially magnified wave curve is shown in Figure 4c. The experimental results show that the organic micro patterns have flexibility instead of rigidity and maintain continuity of arc contours. When the organic micro pattern is deposited on the flexible substrate, the micro pattern deforms with the bending deformation of the flexible substrate. Moreover, the organic micro pattern that produces bending deformation does not destroy the continuity.

### 3.2. Effect of Process Parameters on the Width of Organic Micro Patterns

**Figure 5:**
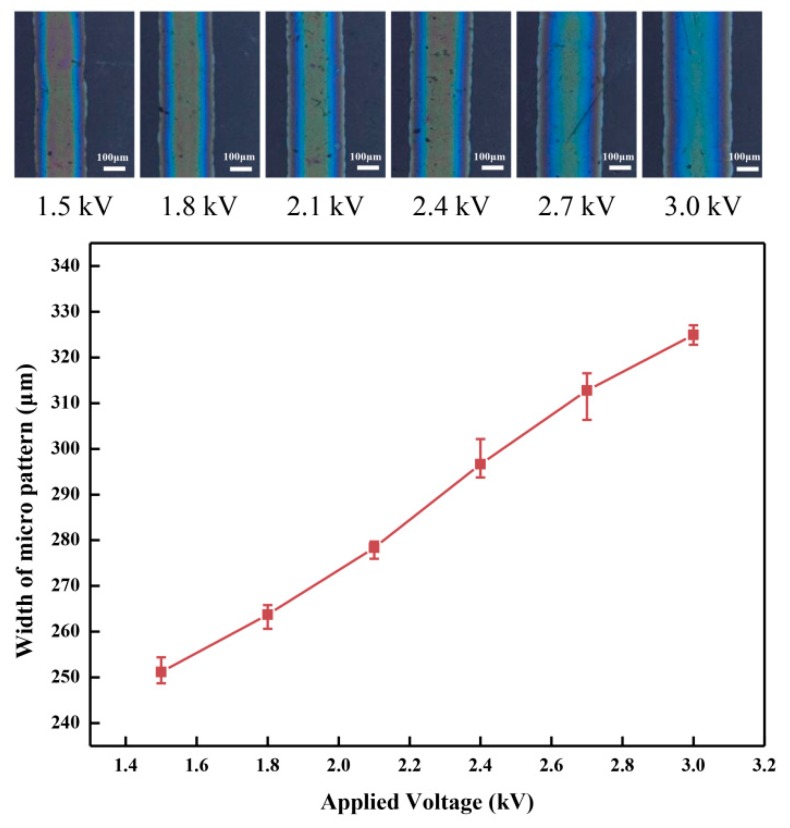
Effect of the applied voltage on the organic micro pattern width.

The applied voltage of the high-voltage power supplier is a key process parameter of the direct-writing micro pattern for the NFEDW method, and ions in the PEDOT:PSS mixed solution generate a downward electric field force under the action of the applied voltage. The fabricated organic micro patterns were observed on the inverted metallographic microscope (DMI3000M, Leica). The average width of the organic micro pattern increased from 251.2 to 325.0 µm with the increase of the applied voltage from 1.5 to 3.0 kV under the conditions of a direct-writing height of 0.35 mm, moving velocity of 10 mm/s for the motion platform, and flow rate of 80 nL/min in the injection system, which are shown in Figure 5. When higher voltage is applied by the high-voltage power supplier, higher axial and radical electric fields are generated, and the organic conductive solution of the mixed PEDOT:PSS materials is subjected to greater electric field force in the NFEWD process. Under the action of larger electric field force, more solution is ejected from the nozzle. When the motion platform has the same velocity and distance, more solutions are pulled out from the nozzle and deposited on the flexible insulating substrate, which leads to the increase of the width of the organic micro pattern. On the other hand, with the increase of the applied voltage, the electric field intensity increases, and this leads to more charges accumulated on the surface of the Taylor cone. The charge density of the direct-writing jet also changes the motion of the jet and the shape of the micro pattern, thus further increasing the width of the organic micro pattern.

**Figure 6:**
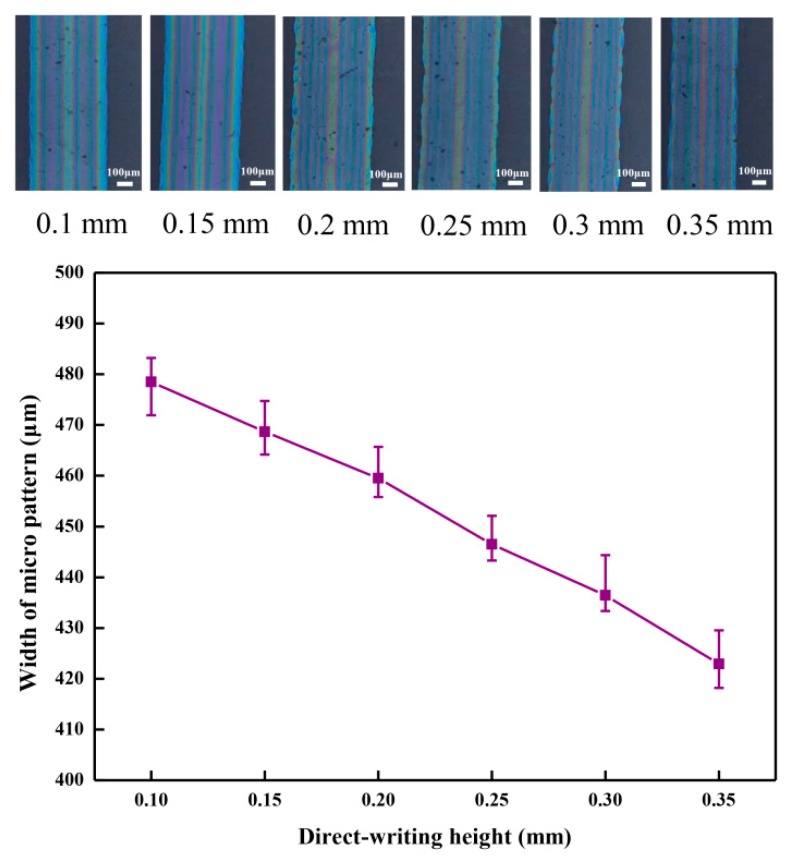
Effect of the direct-writing height on the organic micro pattern width.

The direct-writing height affects the width of the fabricated organic micro pattern when using the NFEDW method. The organic micro patterns were observed on the inverted metallographic microscope (DMI3000M, Leica). The average width of the organic micro pattern decreased from 478.5 to 422.9 µm with the increase of the direct-writing height from 0.1 to 0.35 mm under the conditions of an applied voltage of 2.0 kV, moving velocity of 5 mm/s for the motion platform, and flow rate of 166 nL/min in the injection system, which are shown in Figure 6. The change of the direct-writing height promoted the change of the electric field force on the direct-writing solution of the PEDOT:PSS mixed materials. When the applied voltage was constant, the electric field force on the PEDOT:PSS mixed solution decreased with the increase of the direct-writing height. Therefore, the width of the organic micro pattern decreased with the increase of the direct-writing height.

**Figure 7:**
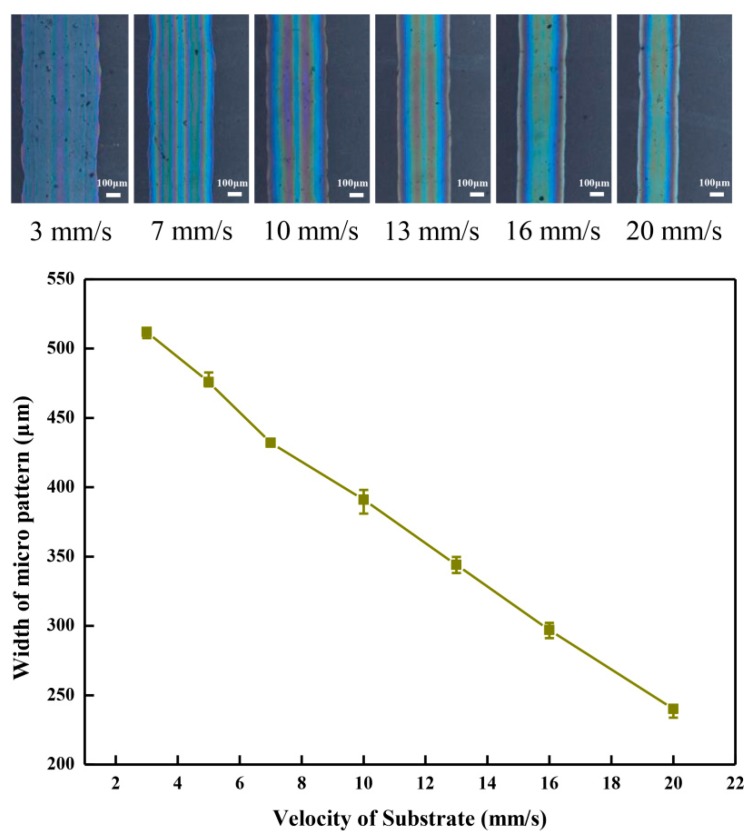
Effect of the velocity of substrate on the organic micro-pattern width.

The moving velocity of the flexible substrate is also an important parameter affecting the width of the organic micro pattern. The organic micro patterns were observed on the inverted metallographic microscope (DMI3000M, Leica). The average width of the organic micro pattern decreased from 512.9 to 297.3 µm with the increase of the moving velocity of the flexible substrate from 3 to 20 mm/s under the conditions of an applied voltage of 2.0 kV, direct-writing height of 0.4 mm, and flow rate of 166 nL/min in the injection system, which are shown in Figure 7. On the one hand, under the same volume of the direct-writing solution, with the increase of the moving velocity of the flexible substrate, the distance of the deposited micro pattern increases, so the width of the organic micro pattern deposited on the flexible substrate decreases. On the other hand, because the PEDOT:PSS mixed solution has certain viscous and surface tension, the moving substrate exerts a tractive force on the direct-writing solution during the movement of the flexible substrate. With the increase of the moving velocity of the flexible substrate, the tractive force increases, and this reduces the width of the organic micro pattern. In addition, increasing the moving velocity of the flexible substrate is helpful to prevent the direct-writing solution from expanding around, which can effectively improve the deposition quality of the organic micro pattern.

**Figure 8:**
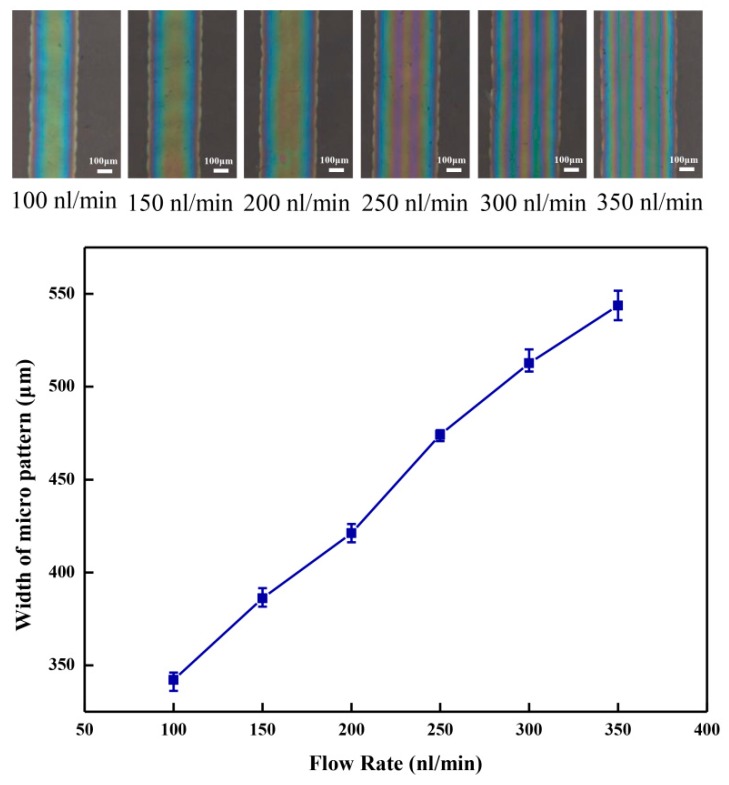
Effect of the flow rate of the injection system on the organic micro pattern width.

The flow rate of the injection system also affects the width of the organic micro pattern in the NFEDW process. The organic micro patterns were observed on an inverted metallographic microscope (DMI3000M, Leica). The average width of the organic micro pattern decreased from 543.7 to 342.1 µm as the flow rate of the injection system decreased from 350 to 100 nL/min under the conditions of an applied voltage of 2.0 kV, direct-writing height of 0.15 mm, and moving velocity of 10 mm/s for the motion platform, which are shown in Figure 8. When the diameter of the nozzle remained unchanged, the change of the flow rate of syringe pump led to the change of the pressure of the injection system. When the flow rate of the injection system decreased, the volume of the PEDOT:PSS mixed solution pulled out from the nozzle decreased under the action of the electric field. Therefore, as the flow rate of the injection system decreased, the diameter of the direct-writing jet decreased, and less volume of the PEDOT:PSS mixed solution was deposited on the flexible insulating substrate in the direct-writing process, which reduced the width of the deposited organic micro pattern. In addition, the direct-writing jet with a smaller radius has larger specific surface area, which accelerates the evaporation of solvent. The experimental results show that the effect of the flow rate of the injection system on the width of the deposited organic micro pattern was more obvious than that of the applied voltage, direct-writing height, and moving velocity of the flexible substrate. Therefore, the direct-writing resolution of the fabricated organic micro pattern can be effectively improved by reducing the flow rate of the injection system or the inner diameter of the nozzle in the NFEDW method.

**Figure 9:**
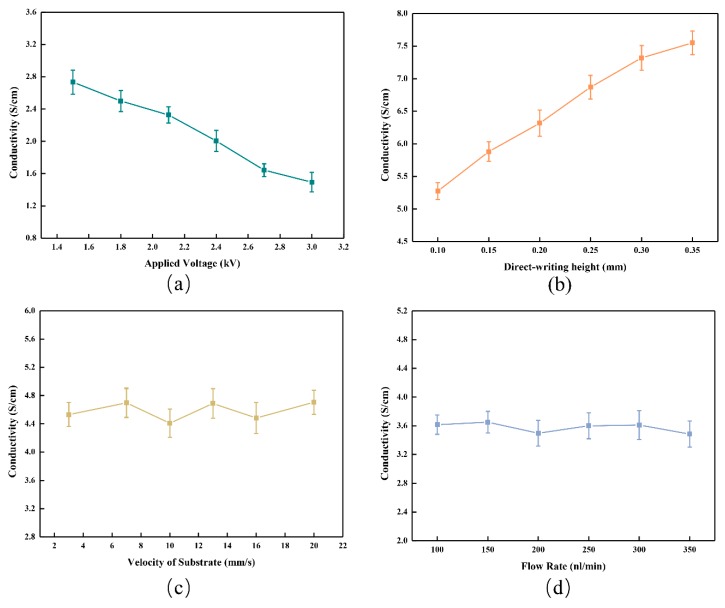
Effect of different process parameters on the electrical conductivity. (**a**) An applied voltage from 1.5 to 3.0 kV, direct-writing height of 0.35 mm, moving velocity of 10 mm/s, and flow rate of 80 nL/min. (**b**) A direct-writing height from 0.1 to 0.35 mm, applied voltage of 2.0 kV, moving velocity of 5 mm/s, and flow rate of 166 nL/min. (**c**) A moving velocity from 3 to 20 mm/s, applied voltage of 2.0 kV, direct-writing height of 0.4 mm, and flow rate of 166 nL/min. (**d**) A flow rate from 100 to 350 nL/min, applied voltage of 2.0 kV, direct-writing height of 0.15 mm, and moving velocity of 10 mm/s.

The electrical conductivity of the untreated PEDOT:PSS is 0.2–1 S/cm [26]. The fabricated organic microcircuit patterns of the natural drying in air at room temperature were tested by a thin film thermoelectric tester (JouleYacht, TRT-100, Wuhan Joule Yacht Science and Technology Co., Ltd., Wuhan, China) at a detection temperature. With the increase of the applied voltage from 1.5 to 30 kV, the conductivity of the fabricated organic micro pattern decreased from 2.7 to 1.49 S/cm, which is shown in Figure 9a. With the increase of the direct-writing height from 0.1 to 0.35 mm, the conductivity increased from 5.28 to 7.76 S/cm, which is shown in Figure 9b. The conductivity of the organic micro pattern remained basically unchanged with the increase of the moving velocity for the substrate and the flow rate for the injection system under the condition where the three other process parameters remained constant, respectively, which are shown in Figure 9c,d. Therefore, the applied voltage and the direct-writing height have an effect on the conductivity. With the increase of the applied voltage, the electric field force increased, and this led to the increase of the organic micro-pattern width (Figure 5). Under the condition where the three other process parameters remained constant, the conductivity decreased with the increase of the organic pattern width. With the increase of the direct-writing height, the electric field force decreased, and this led to the decrease of the organic micro-pattern width (Figure 6). The decrease of the fabricated pattern width led to the increase of its conductivity under the conditions where the three other process parameters remained constant.

**Figure 10:**
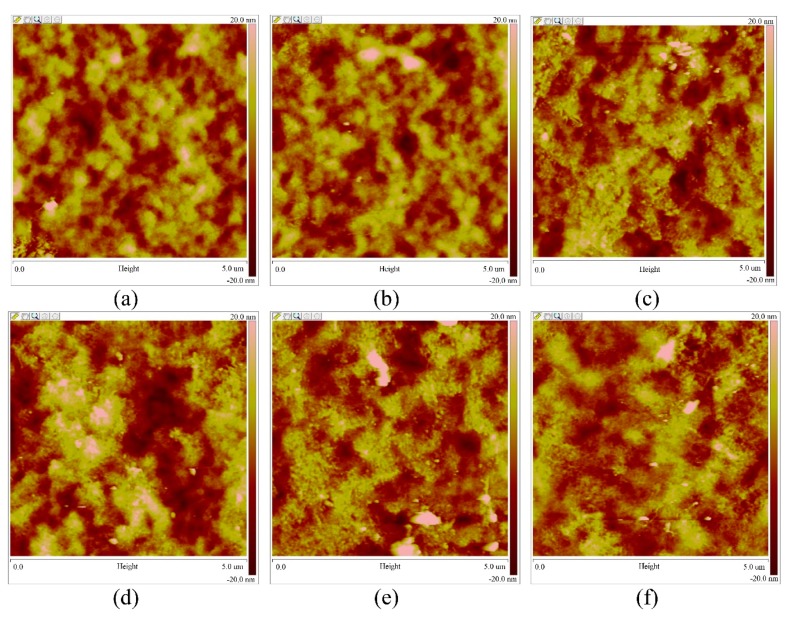
The atomic force microscopy (AFM) images of the direct-writing micro patterns with different process parameters. (**a**) A flow rate of 100 nL/min. (**b**) A flow rate of 150 nL/min. (**c**) A flow rate of 200 nL/min. (**d**) A flow rate of 250 nL/min. (**e**) A flow rate of 300 nL/min. (**f**) A flow rate of 350 nL/min.

**Figure 11:**
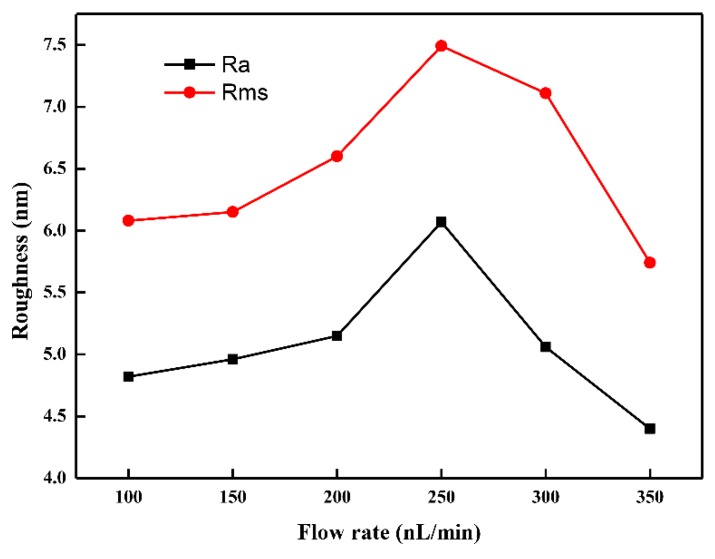
The roughness of the deposited micro pattern. (Rms is the root mean square average of the height deviations taken from the mean data plane; Ra is the arithmetic average of the absolute values of the surface height deviations measured from the deposited micro pattern).

The atomic force microscopy (AFM) images of the deposited micro patterns are shown in Figure 10. When the flow rate of the injection system increased from 100 to 350 nL/min under the conditions of an applied voltage of 2.0 kV, direct-writing height of 0.15 mm, and moving velocity of 10 mm/s for the motion platform, surface microstructures developed, as shown in Figure 10. The roughness of the deposited micro patterns is shown in Figure 11. By analyzing Figure 10, it can be determined that the internal structure and morphology of the micro patterns are very similar. When the other three parameters remained unchanged, the effect of the flow rate variation on the micro pattern was very small. By analyzing Figure 11, it can be determined that the roughness of the organic micro patterns changed very little with the increase of the flow rate of the injection system under the condition where the three other process parameters remained constant.

## 4. Conclusions

This study shows that it is possible to fabricate the micro pattern of PEDOT:PSS organic conductive polymer material on a flexible insulating substrate by using the near-field electrohydrodynamic direct-writing method. The effect of the process parameters on the width and the conductivity of the fabricated organic micro pattern were studied. From the results of this study, the following conclusions can be drawn:

(1) Organic micro patterns of various shapes, such as the curve of the second-order self-similar structure, helical curve, and wave curve, can be fabricated on the flexible insulating substrate with the PEDOT:PSS organic conductive polymer material as the direct-writing solution by using the near-field electrohydrodynamic direct-writing method.

(2) The process parameters, such as the applied voltage, direct-writing height, moving velocity of the flexible substrate, and flow rate of the injection system, affect the width of the organic micro pattern in the near-field electrohydrodynamic direct-writing process. Under the condition that the other process parameters remain constant, the width of the organic micro pattern increases gradually with the increase of the applied voltage, and the width of the organic micro pattern decreases gradually with the increase of the direct-writing height. Under the condition that the three other process parameters remain constant, the width of the micro pattern decreases with the increase of the moving velocity of the flexible substrate, and the width of the organic micro pattern decreases gradually with the decreases of the flow rate of the injection system.

(3) The effect of the flow rate of injection system on the width of the deposition organic micro pattern is more obvious than that of the applied voltage, direct-writing height, and moving velocity of the flexible substrate. The direct-writing resolution of the fabricated organic micro pattern can be effectively improved by reducing the flow rate of the injection system or the inner diameter of the nozzle in the near-field electrohydrodynamic direct-writing process.

(4) With the increase of the electric field intensity, the conductivity of the fabricated organic micro pattern decreases, and the effect of the moving velocity and the flow rate on the conductivity is small under the condition that the three other process parameters remain constant.

## Figures and Tables

**Table 1:** Comparison of the main micro-patterning techniques. EHD—electrohydrodynamic; NFEDW—near-field EHD direct-writing.

	Lithography	Imprint	EHD Inkjet Printing	NFEDW
Cost	Extremely high	Secondary	Low	Low
Efficiency	Low	High	High	High
Temperature	High temperature and high pressure	Secondary	Unlimited	Unlimited
Masking	Yes	No	No	No
Resolution	Extremely high	High	High	High
Compatibility of material	Poor	Poor	Good	Good
Positioning accuracy	High	Secondary	Poor	High
Environmental requirement	Purification	Secondary	Low	Low
Process step	Multistep	Multistep	Single step	Single step
